# OBET: On-the-Fly Byte-Level Error Tracking for Correcting and Detecting Faults in Unreliable DRAM Systems

**DOI:** 10.3390/s21248271

**Published:** 2021-12-10

**Authors:** Duy-Thanh Nguyen, Nhut-Minh Ho, Weng-Fai Wong, Ik-Joon Chang

**Affiliations:** 1 Department of Electronic Engineering, Kyung Hee University, Yongin-si 17104, Korea; dtnguyen@khu.ac.kr; 2 Department of Computer Science, National University of Singapore, Singapore 117418, Singapore; minhhn@comp.nus.edu.sg (N.-M.H.); wongwf@nus.edu.sg (W.-F.W.)

**Keywords:** DDR5, on-die ECC, memory architecture, memory management, DRAM chips, error correction codes, fault diagnosis, failure analysis, semiconductor device reliability, availability, semiconductor device testing, debugging

## Abstract

With technology scaling, maintaining the reliability of dynamic random-access memory (DRAM) has become more challenging. Therefore, on-die error correction codes have been introduced to accommodate reliability issues in DDR5. However, the current solution still suffers from high overhead when a large DRAM capacity is used to deliver high performance. We present a DRAM chip architecture that can track faults at byte-level DRAM cell errors to address this problem. DRAM faults are classified as temporary or permanent in our proposed architecture, with no additional pins and with minor DRAM chip modifications. Hence, we achieve reliability comparable to that of other state-of-the-art solutions while incurring negligible performance and energy overhead. Furthermore, the faulty locations are efficiently exposed to the operating system (OS). Thus, we can significantly reduce the required scrubbing cycle by scrubbing only faulty DRAM pages while reducing the system failure probability up to 5000∼7000 times relative to conventional operation.

## 1. Introduction

In modern computer systems, a larger dynamic random-access memory (DRAM) chip capacity is required for high performance. With such a trend, the reliability of DRAM becomes more critical to delivering dependable system operations. The conventional approach to assure the reliability of DRAM is to characterize the problems of DRAM chips when they are tested. Then, the characterized problems are addressed by some post-silicon techniques, such as replacing problematic rows and columns. However, some reliability problems cannot be handled by these techniques. For instance, in scaled DRAM devices, row hammering attacks [1] are a major security issue that can significantly degrade the reliability of DRAM. In current computer systems, the occurrence of row hammering is predicted and prevented by using online techniques [2,3]. Other reliability problems are unpredictable; DRAM failure due to cosmic radiation is one example. High-energy neutrons from space are known to cause single-event upsets in DRAM, flipping single or multiple bits [4,5]. Fortunately, such errors are transient and recoverable and hence are called *soft errors*. As reported in [6,7,8,9,10,11], soft errors are important concerns for reliability improvement, with the solution for static RAM (SRAM) proposed in [6,9] and the solution for latch proposed in [8,11]. To prevent soft errors in DRAM devices, architectural techniques based on *error correction codes* (ECCs), such as single error correction and double error detection (SECDED) and Chipkill [12], are widely used in modern computer systems. A popular choice for an ECC is the SECDED code. In the SECDED architecture, DRAM control units perform error correction by using a *rank-level ECC*, where the parity bits required for the ECC are stored in additional DRAM chips. Software-level recovery techniques such as checkpoints and snapshots of DRAM devices are used to address multibit errors. The problem with these techniques is that the performance and energy overhead are significant. The key premise of the SECDED-based DRAM system is that the probability of multibit error cases is much lower than that of single-bit error cases; hence, software recovery is infrequently employed.

Unfortunately, with the scaling of DRAM technologies, the bit error rate (BER) of DRAM chips deteriorates, threatening the key premise of the SECDED-based DRAM system. An industrial report shows that in sub-20 nm technologies, DRAM cells suffer from poor retention [13]. Furthermore, in scaled DRAM chips, variable retention time becomes significant. The combination of poor retention and variable retention time makes it challenging to ensure the integrity of data stored in DRAM. Additionally, neutrons due to cosmic rays cause recoverable soft errors and nonrecoverable hard errors in scaled DRAM devices. DRAM faults due to cosmic radiation have been identified in DRAMs delivered by flights since the neutron flux is significantly higher at high altitudes than at sea level [5,14,15,16]. This has motivated the need for on-die ECCs, now a part of the JEDEC standard since LPDDR4 and DDR5 [17,18].

One may expect that by employing both on-die and rank-level ECCs, i.e., a two-level ECC, the reliability of DRAM chips will be further improved. However, in DDR5, on-die and rank-level ECCs operate independently. Proposals such as XED [19] and DUO [20] adopt the requirement constraint of 6.25∼12.5% overhead for on-die ECC’s parity bits [21], they enhance the error correction capability for only one level of ECC with strong ECC that is computationally heavy or utilize the parity bits of on-die ECCs as auxiliary information for the rank-level ECC, and additional data transfers from the chips are required. All state-of-the-art works are not efficient in modern DRAM architecture due to requirements of high performance, which may break the JEDEC specification.

Hence, this work presents an on-the-fly byte-level error tracking scheme, called OBET, which is a post-silicon method for detecting and correcting faults in modern DRAM architectures that support on-die ECCs. Our proposed scheme provides a strong error correction capability with minimal modifications to the current DRAM architecture. It is well known that in DRAM, an on-die ECC is implemented as a single error correction code due to the tight area constraint of DRAM. If a single error occurs in the same word as an existing hard error, it cannot be addressed by a single error correction code. With the scaling of technology, both hard and soft error rates tend to increase. Then, the probability that the above case occurs will also increase. We consider that our byte-level error tracking and error type classification methods effectively handle this problem. Notably, the bit-level information regarding DRAM errors reveals much critical information. However, our technique exposes only byte-level error information, whereas the exact bit error locations are not exactly known, mitigating the security issue to a certain degree. OBET can fully comply with the modern DRAM architecture and employs both on-die and rank-level ECCs. Furthermore, an ECC *error check and scrub* (ECS) mode is required in the new JEDEC specification of DDR5. This incurs a significant overhead. However, our OBET can significantly reduce it. Our key contributions can be summarized as follows:

- We propose an OBET architecture that provides runtime byte-level error tracking without additional DRAM input and output pins. OBET achieves this by exploiting a small number of pins that are not used during data transfers.
- We develop a memory fault management scheme based on our OBET architecture, where permanent fault pages are efficiently diagnosed and retired. Our proposed scheme targets both soft and hard failures, such as those caused by cosmic rays in scaled DRAM technologies.
- In OBET, the overhead of scrub operations, which are required to prevent DRAM error accumulation, is significantly reduced. This allows us to efficiently regulate the probability of multiple-bit errors in a word, for which rank-ECC cannot provide correction.

This paper is organized as follows. In Section 2, we review the current DRAM architecture and explain some components that need to be modified in the OBET architecture. Next, we briefly discuss in detail the major DRAM challenges and motivation of our works in Section 3. The details of our OBET architecture are discussed in Section 4 and Section 5. We then compare OBET-based schemes with some state-of-the-art works in terms of reliability, performance, and energy in Section 6.1. Next, Section 7 shows how to adopt OBET for more efficient scrubbing methods for current DRAMs. Finally, we conclude our work in Section 8.

## 2. Preliminary Background

### 2.1. DRAM Organization and Operation

**Figure 1:**
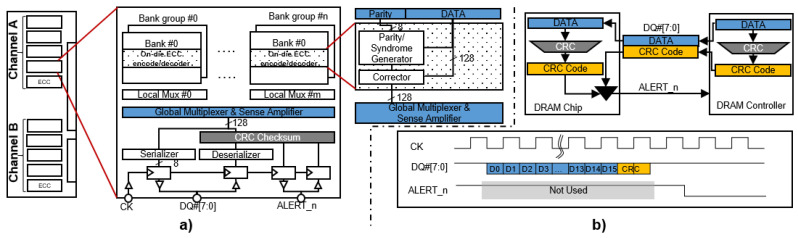
Summary of the background on the DRAM architecture and on-die ECC: (**a**) the hierarchical organization of DRAM and the position of the on-die ECC in the DRAM device and (**b**) a functional description of a **ALERT_n** pin.

Our work is based on the architecture of DDR5 DRAMs [18]. DRAMs usually come in the form of a dual in-line memory module (DIMM) such that multiple DRAM chips are placed on one or two sides of a printed circuit board (PCB). These chips are hierarchically organized into groups of {channel, rank, bank group, bank, row, column, DRAM word}. One DDR5 DRAM channel, shared mainly by two ranks, has 32 data-bus lines for data. DDR5 DRAMs that support rank-level ECC have an additional 8 data-bus lines for rank-level ECC parity bits. A rank of DDR5 DRAMs is made up of multiple DRAM chips, each having 8 or 4 data-bus lines for the ×8 or ×4 DRAM chip configuration, respectively. The burst length of DDR5 DRAM is sixteen. Therefore, the burst data size is 64 bytes (=32 data bits × the burst length). This implies that with a single burst read or write command, 64∼128 bits of data (4/8 bits × burst length) are read from or written to a DRAM chip. Therefore, we assume that the number of data bits for a codeword of an on-die ECC is 128 bits. We further assume that single-error correction (SEC) is used for the on-die ECC and that the number of parity bits is 8 bits. Such a configuration, as shown in Figure 1a, is used throughout this work, where the 16 banks (8 bank groups and 2 banks per bank group) has its own on-die ECC. When a read command is issued, the 128-bit data and 8-bit parity are sensed from local sense amplifiers and sent to the on-die ECC decoder. When a single-bit error is detected, the on-die ECC decoder corrects the error and sends the corrected data to the serializer, where the corrected 64∼128-bit data are split over the 16 bursts. Here, the on-die ECC does not update the erroneous DRAM cell information, and hence, the error stays in the DRAM array. Using the Hamming code, eight parity bits can detect the error for 255 bits. In implementing the hamming correction code, a syndrome block provides the error position in 255 bits. According to DDR5, we only use eight parity bits to protect 136 bits in DDR5. Hence, any provided error positions out of 136 can be considered as multiple bit errors. Therefore, mostly ∼47% multiple errors can be detected. If there is no error or a double-bit error detected (∼47% detecting chance) by the on-die ECC decoder, the data are sent to the serializer without modification. In the case of a double-bit error, the erroneous data are sent to the memory controller. Again, the errors are not corrected in the DRAM array.

### 2.2. Read-Modify-Write

With a burst write command, the 8-bit parity is computed from the 128-bit data received and written to DRAM cells internally. However, for some write commands, only a few 4∼8-bit data are transferred from the memory controller, a 128-bit read operation needs to be first performed internally, and then the new 8-bit data are updated. Based on the updated data, a new 8-bit parity is computed by the on-die ECC encoder. This operation is referred to as ‘read-modify-write’. As reported in [22], the write timing is four times larger than the read timing due to the time consumed by the ‘read-modify-write’ operation. However, read operations usually dominate over write operations in most programs. In a real system, the write operations are not timing critical because the support of multilevel caching and the computing unit do not need to stall to wait for the writing operation to be finished [23,24]. Furthermore, to make the on-die ECC encoding in a bank while decoding in another bank simultaneously, each bank will have its own on-die ECC, as shown in Figure 1a.

### 2.3. Function of **ALERT_n** Pin

As defined in the JEDEC standard [18,25], ALERT_n pins are used to report errors in a transmission channel. The detailed function of ALERT_n is shown in Figure 1b. To verify the data transmission channel, a cyclic redundancy code (CRC) is added to transmitted data. When the CRC detects a transmission error, the corresponding ALERT_n pin goes low after the transmitted data. The ALERT_n pins are rarely used during normal DRAM operations because performance is significantly degraded. Furthermore, the ALERT_n pins are also used as the input in the connectivity mode (CT) as defined in JEDEC. Hence, ALERT_n pins are bi-directional for both input and output purposes. In our OBET architecture, we can exploit the ALERT_n pins for reporting the errors between the on-die ECCs and the corresponding DRAM controller. This can be achieved by partial modification of the DRAM circuits. The ALERT_n pin is not designed as the high-speed signaling pin. Using and modifying it for for our purpose require extra overhead. However, the overead is considerably acceptable when it helps to significantly improve the DRAM reliability.

### 2.4. DRAM Faults

Based on the discussion in recent works [26,27], there are three types of DRAM faults: (1) transient faults, (2) intermittent faults, and (3) permanent faults. Transient faults occur randomly throughout the entire DRAM array. Transient faults do not result from DRAM cell physical damage and can be recovered by rewriting the corresponding erroneous DRAM cells. Variable retention times (VRTs), row hammering, and soft errors due to cosmic rays introduce transient faults. Unlike transient faults, intermittent and permanent faults occur when DRAM cells are physically damaged. The difference between the two is the degree of damage. If the damage is not substantial enough, then the damaged cell can still produce the correct result most of the time but fails repeatedly every so often, called an intermittent fault. On the other hand, permanently damaged cells do not work at all.

Therefore, the locations of permanent and intermittent faults are fixed and cannot be repaired by simply rewriting the corresponding DRAM cells. Permanent faults always result in errors, while the errors due to intermittent faults depend on external conditions, making them hard to identify with mere testing. In scaled DRAM technologies, cosmic rays cause both permanent and intermittent faults [14,27], implying that the number of faulty cells increases whenever DRAM products are delivered through aviation. Our on-the-fly error tracking, an efficient fault diagnosis, and management scheme termed OBET addresses this problem.

### 2.5. DDR5 ECC Transparency and Scrubbing

In DDR5, JEDEC defines a scrubbing mode called ECS to prevent error accumulation in DRAMs. The ECS mode must be performed at least once every 24 h for the entire DRAM chip. However, it can accommodate only single-bit errors due to transient faults. In a scrubbing cycle, the on-die ECC reads and checks a codeword (data plus ECC bits). If a single-bit error is found, the error is corrected. Then, the corrected codeword is written back to DRAM cells, completing a scrubbing cycle. Two ECS modes are defined in DDR5: automatic and manual ECS modes. In the automatic ECS mode, the memory controller needs to issue periodic REFab commands or periodically enter ‘self-refresh’ mode. DRAM chips are interrupted during the automatic ECS mode. For the manual ECS mode, the memory controller issues the command to start the mode, generating the following internally self-timed command sequence: ACT, RD, WR, and PRE. The manual ECS mode can be performed without interrupting DRAM chips. Both ECS modes rely on the internal ECS address counter to access the memory array rows, which cannot be controlled by the memory controller. Therefore, the output of the internal ECS address counter sequentially increases. To scrub a DRAM chip, entire rows in the DRAM chip should be scanned. This results in considerable power and performance overhead. OBET significantly improves this limitation by selectively performing scrubbing only for faulty rows.

## 3. Modern DRAM Issues and Motivation

### 3.1. Technology Scaling and Cosmic Rays

DRAM is the most frequent faulty component in modern computer systems and requires regular replacement in data centers [14,28]. In addition, the amount of DRAM consumed by current software is significantly growing, causing DRAM chip densities to increase over time. To adapt to DRAM’s increasing density trend, hardware designers have made smaller DRAM cells. These cells are easy to suffer hard faults due to process variation in lower process node. They also require a shorter retention time which making these cells more sensitive to the environment. Thus, smaller DRAM cells can undergo significantly more errors during delivery and operation in servers due to the short retention time and cosmic rays, as reported in [14,26]. These weaker cells are expected to become the major issue of systems in the near future, especially in single cell errors. To overcome such errors, JEDEC [18] requires using the on-die ECC to correct single errors only without correcting the errors in DRAM devices because of the critical timing. When the errors accumulate, the system crashes when the ECC cannot correct multiple errors.

### 3.2. Row Hammering

Kim et al. [1] introduced the potential of row hammering attacks due to a large number of accesses on adjacent rows. As a result, row hammering has become the major security issue that threatens the system’s reliability. Recently, refs. [1,29,30] showed that actual errors are needed to study and prevent these attacks. Row Hammering errors can be referred as soft errors. As suggested in [1], increasing the refresh rate to reduce the errors become the key solution. JEDEC also pays attention to solve such row hammering problem by providing the error threshold registers and adaptively adjusting the refresh rate of DRAM. However, a study in [31] has shown that the neutron rays easily cause hard errors in modern DRAM devices. Such hard errors are accumulated in the lifetime of DRAM devices and can blend with row hammering errors to becomes a complicated issue. It is hard to diagnose the main error source in this case. Hence, exposing the error and specifying the error type are critical. However, these errors are hard to detect or collect with the on-die ECC in the DDR5 architecture because the on-die ECC automatically corrects them before reading data out. Recently, Ref. [32] provided a method to predict the on-die ECC functions that help debug the error in DRAM. However, this method cannot improve the reliability of DRAM, and it is difficult to provide exact error locations in DRAM. Therefore, the errors are not exposed to the outside, making it hard to control or diagnose their positions.

### 3.3. State-of-the-Art Works Related to the On-Die ECC

To improve the reliability of DRAM, Son et al. [33] attempted to cache the word error location by using on-chip SRAM. However, according to technology scaling, a single error tends to be significant. Hence, this technique becomes inefficient in modern DRAM (especially in on-die ECC design), incurring a large overhead in DRAM, while [34] reported that even an overhead 1% area increment in DRAM is too expensive.

Recently, Nair et al. [19] noted that the hidden errors inside the DRAM chip due to on-die ECC reduced the reliability of the system. In XED [19], the on-die ECC performs only error detection in DRAM chips, and rank-ECC is used to correct the error. XED becomes weak in correcting multiple errors due to technology scaling. DUO [20] used a stronger Reed-Solomon (RS) code with a longer block code. However, hardware decoding is a polynomial problem, while DRAM access is timing critical, and an additional burst is also required. Hence, DUO breaks the JEDEC standard [17,18]. PAIR [35] maximized the utilization of RS code and reduced the overhead of ‘read-modify-write’, but the error information still cannot be exposed to the external system.

The above works made significant accomplishments to enhance the reliability of DRAMs. However, several problems still exist. For instance, XED fails to address the case of single-bit errors occurring simultaneously to two DRAM chips in a rank. Both DUO and PAIR use RS code, which is computationally burdensome. Hence, it is difficult for error correction scenarios based on DUO or PAIR to meet the strict latency constraint of DRAM. Furthermore, XED, DUO, and PAIR require the system to access DRAM as a block (normally 512 bits). Therefore, they cannot work for a single burst (32–64 bit). Note also that the above techniques and conventional ECC techniques cannot address errors stored in DRAM and suffer from the accumulation of memory errors. In the DDR5 architecture, the use of the on-die ECC is dependent on rank-ECC, and the errors are not exposed to rank-ECC. Hence, we recognize the possibility of providing cooperative two-level ECC schemes, and we utilize both on-die and rank-level ECC to further improve the reliability.

### 3.4. Motivation

Overall, the major challenges of modern DRAM can be summarized as follows:

- DRAM is the most frequently failing component in hardware failures [28].
- The amount of required DRAM continues to grow, while technology scaling causes more errors. Short retention times, cosmic ray effects, and row hammering attacks have become severe issues in modern DRAM.
- In the DDR5 architecture, errors that occur in DRAM are silently masked by the on-die ECC. Because errors are not corrected, it is difficult to diagnose and study their behavior. It is necessary to expose the error information to the system.
- State-of-the-art works are still inefficient on DDR5.

These problems are essentially solved in this work. We provide a new architecture termed OBET that can fully adapt to the DDR5 architecture that minimizes errors. The details are discussed in the next section.

## 4. OBET Architecture

### 4.1. System Overview

**Figure 2:**
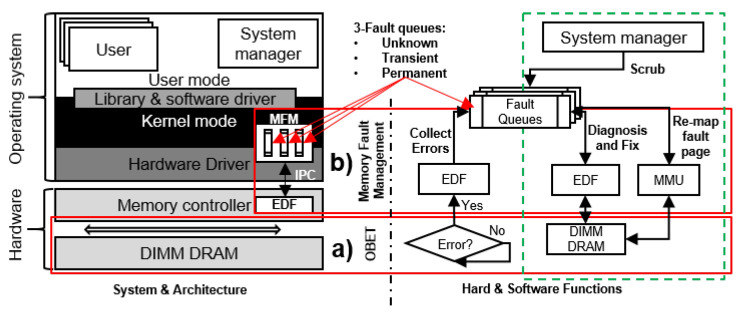
Summary of OBET’s contribution and a full system overview, from the devices to the operating system: (**a**) the position and operation of OBET and (**b**) the position and operation of memory fault management.

OBET is summarized in Figure 2. By enabling a communication channel between the memory controller and on-die ECCs, OBET tracks the errors of DRAM chips at the byte level. Based on the tracked error information, OBET builds a list of faulty pages. Mcelog [36] is currently providing the memory handling method in OS. However, with the requirement of a new DDR5 architecture, Mcelog no longer works. We then propose OBET’s *memory fault management* (MFM) module in the operating system that can replace Mcelog. We developed an open-source prototype of MFM for Linux. In the MFM, the types of detected memory faults are classified. Pages with permanent or intermittent faults are retired to prevent possible system failures due to corrupted data. We further describe the MFM module in Section 5. One of the major components of OBET is OBET scrubbing, as described in Section 7.

To reduce scaling faults, the JEDEC standard [18] requires the system to perform ECS mode at least once per 24 h for all DRAM devices. This scrubbing increases the downtime of servers in the data center. However, with the proposed architecture, OBET scrubs only the listed faulty pages. Thus, OBET significantly reduces the power and performance overhead of scrubbing. Regardless of whether a rank-level ECC is utilized, the overall architecture of OBET remains the same. Hence, we describe OBET without a rank-level ECC in this section.

### 4.2. DRAM Architecture for OBET

#### 4.2.1. Generating a Byte-Error Flag

**Figure 3:**
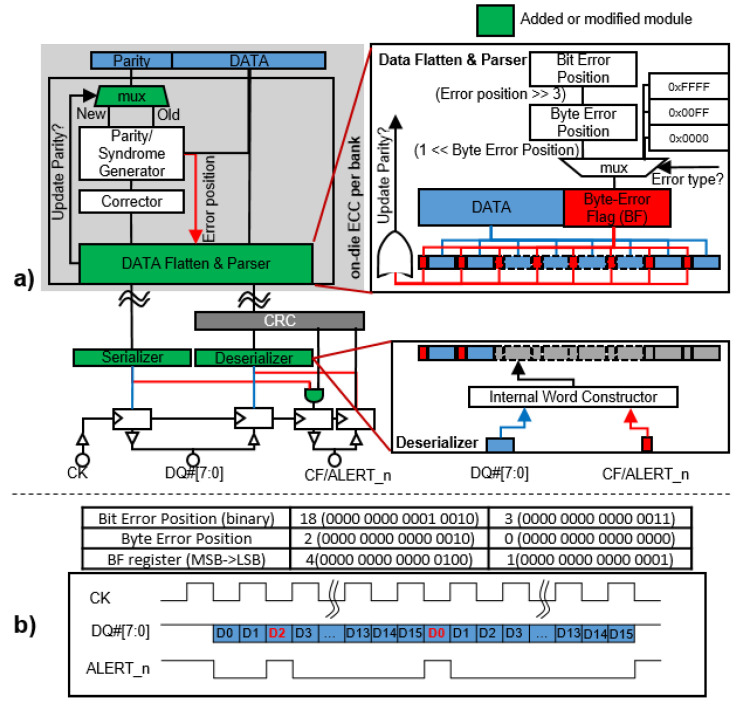
A description of the byte-error tracking module in the DRAM architecture: (**a**) the byte-error flag DRAM architecture and (**b**) an example of byte-error flag timing, replying on the 18th bit and 3rd bit of the first and second DRAM words (note: the green modules are our modifications in OBET).

In DDR5 DRAM, on-die ECCs correct errors of the read data; however, they do not fix the corresponding faulty cells. Furthermore, there is no way that on-die ECCs can tell the external world whether errors occurred in the read data. Under such circumstances, the system cannot execute the run-time repair of faulty cells, leading to error accumulation. In OBET, we propose a byte-error flag format to expose the error information recognized by on-die ECCs to the system. Then, the system finds the most effective way to handle the error. To generate the byte-error flag, we modify the architecture of the on-die ECC encoder/decoder, as shown in Figure 3a. The signals to indicate the positions and the types of errors are produced by the parity/syndrome generator and sent to the *data flattened and parse* (DFP) module. Here, a *byte-error flag* (BF) register is defined as follows.

(1) $$BF=\left\{\begin{array}{cc} 0 & \mathrm{no}\;\mathrm{error}\\ 0\times 00FF & \mathrm{multiple-bit}\;\mathrm{error}(∼47\%\mathrm{detecting}\;\mathrm{chance}),\;\mathrm{DUE}\\ 0\times FFFF & \mathrm{single-bit}\;\mathrm{parity}\;\mathrm{error}\\ 1\ll (\mathrm{bit}\;\mathrm{error}\;\mathrm{position}/8) & \mathrm{single-bit}\;\mathrm{data}\;\mathrm{error}. \end{array}\right.$$

Assuming the popular SEC(136,128), the bit error position ranges from 0 to 127 for a single-bit error. BF is set to 0×FFFF for a single-bit parity error when the bit error position ranges from 128 to 135. As discussed in Section 2, single error correction code also can provide only ∼47% chance of detecting multiple-bit errors. When a multiple-bit error is detected and uncorrected, so-called DUE, we assign BF = 0×00FF in Equation (1). For example, suppose the bit error position is 34 and the byte error position is 4 (34/8 = 4). The BF register value is 1<<4 = ‘10 000’ in binary. By merging each bit in the BF register with a 16-bit fragment from the original data, we have an error flag for each outgoing byte of the DRAM device. The one-bit byte-error flag can be sent to the corresponding memory controller via the ALERT_n pin by simply performing an “AND” operation with the output of the normal CRC module. The internal DRAM error can occur either in data or parity. Errors in the data are easily tracked by the memory controller by observing the ALERT_n pin. However, it is difficult to track parity errors; thus, we propose that all BF bits are able to report these errors. The BF can be used as the control signal, exploited to diagnose the error type, to prevent parity updating. This can be simply implemented by adding a multiplexer that selects either the old parity or the new parity based on the BF control signal.

#### 4.2.2. On-the-Fly Byte-Level Error Tracking

Our OBET only comprises one shifter and a few multiplexers. We use a low-power 65 nm complementary metal-oxide-semiconductor (CMOS) to estimate the hardware overhead of OBET. The result shows that 36.8 μm${}^{2}$ (1.6%) hardware area is added and the power is increased by a mere 0.045 μW (3%) compared to the standard on-die ECC encoder/decoder module. As discussed in Section 2, DRR5 has 16 banks (typically, eight bank groups with two banks per bank group) the total OBET power is consummated 0.72 μW. Compared to the total power of DRAM chip [37], the power overhead increment of 16 on-die ECC is less than 0.01% of the entire DRAM power. DRAM vendors have announced 1z technology for the main DRAM products recently, referring to 15 nm CMOS. As well known that a lower process mode will give the lower power and better performance. Even though we use the 65 nm CMOS to evaluate the power consumption of OBET, it is highly certain that the power overhead is almost negligible for the whole DRAM chip. Furthermore, connecting DRAM banks to the global IO pads also requires additional byte-error flag wires, which have an additional overhead of approximately 6.25% (8 error flag bits for 128 data bits). However, the wiring overhead can be considered as negligible in the context of the whole DRAM (several gigabyte of DRAM cells).

As shown in Figure 3a, in a read operation, the 16-bit BF is transmitted to the corresponding DRAM controller through the ALERT_n pin. When a single-bit error is observed in the data, only one byte has the flag raised. When there is a parity error, all BF bits are raised. For example, the output with the data in Figure 3b indicates that the errors occur in the third byte of data burst #0 and the first byte of data burst #1.

Our design minimizes secret information leakage and hardware modification. Because the bandwidth is the most critical problem in DRAM design, DRAM designers try to extend the data bus instead of the command bus to improve the throughput. When using the data bus to send error information, the data and additional data are sent to the memory controller twice. In this case, the performance is significantly reduced, especially since new versions of DDR always prioritize performance. Hence, we modify the unused ALERT_n pin to provide only byte-error information at runtime since this technique is more efficient than using the command bus or data bus.

## 5. Memory Fault Management (MFM)

In this section, we describe the MFM mechanism that can track memory fault information. This is a module of the operating system (OS) kernel, as shown in Figure 2.

### 5.1. Error Diagnostic and Fixing (EDF) Module

**Figure 4:**
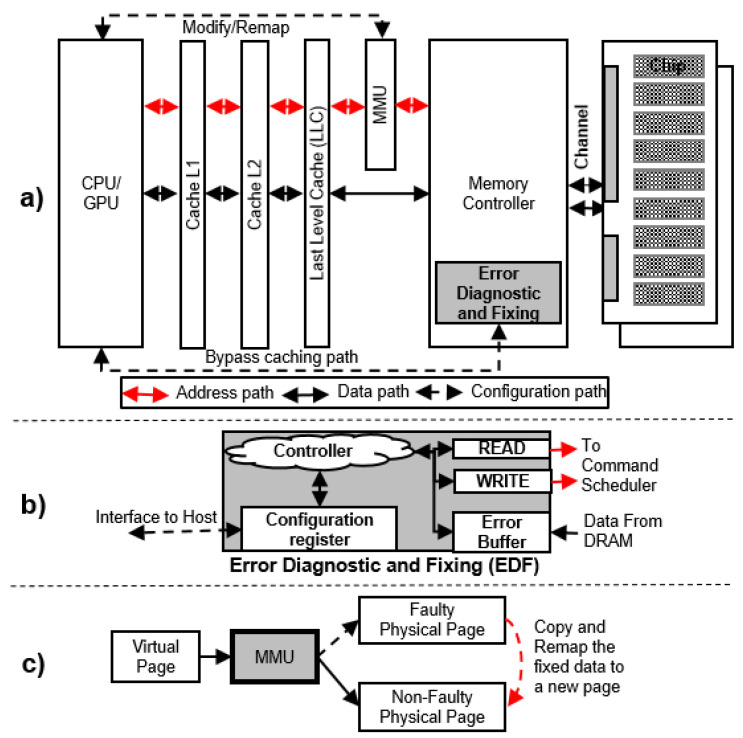
A description of the memory fault management architecture: (**a**) the overall data and control flow in the memory fault management architecture, (**b**) the details of the EDF module, and (**c**) the remapping flow for faulty pages.

The typical computer architecture has multiple-level caches, as shown Figure 4a, where the OS cannot directly access DRAM data. Under such circumstances, the OS cannot efficiently diagnose and fix DRAM errors. Hence, we present an EDF module located inside the memory controller. The EDF is directly configured from the host, which can be implemented with a moderate hardware cost of approximately 64 byte SRAM, the catch-up error buffer that stores all cases as described in Section 5.2.2. Due to the EDF, the system performance is not significantly degraded when the OS manages the faults in DRAM. With the support of the EDF, our MFM provides three services that handle DRAM faults: (1) collecting faulty DRAM pages, (2) diagnosing and fixing the faults, and (3) remapping permanent faulty pages to nonfaulty pages. Their details are discussed in the following subsections.

### 5.2. Operation of the MFM Module

As mentioned above, there are three major operations provided by our MFM: (1) collecting errors, (2) diagnosing and fixing errors, and (3) remapping faulty pages. The details are discussed as follows.

#### 5.2.1. Collecting Errors

This section reports the method by which EDF takes errors from the main memory and sends notification of the corresponding fault types to the fault queues of our MFM. As visualized in Figure 2, we define three fault queues: (1) *transient*, (2) *permanent*, and (3) *unknown*. We implement three error queues to store errors due to the three types of DRAM faults aforementioned in Section 2.4. The OS uses the three fault queues to categorize the errors reported from the EDF module. Many factors affect the sizes of fault queues (i.e., vendor dependence, PVT (process, voltage and temperature) variations, and the altitude of the data center where the DRAM devices are placed). The number of errors may vary, and it is hard to predict this value. Hence, we implement such queues in the OS, where the sizes of fault queues are flexible and are dynamically considered at runtime by the OS.

When an error is reported from the DRAM via BFs, this error is first caught by the EDF module, where all reported errors are first regarded as *unknown*. The details are summarized in Algorithm 1, where the corresponding OS function and EDF procedure are described. The *unknown* faults are classified into *transient* or *permanent* faults by our error diagnostic and repair procedure, discussed in Section 5.2.2. Note that in Section 2.4, we categorize DRAM faults as (1) transient, (2) intermittent, or (3) permanent faults. The errors due to transient and permanent faults are pushed to *transient* and *permanent* fault queues, respectively. The faults due to intermittent faults have both transient and permanent characteristics. We need to observe the fault queues for a long time when the system is running. Then, we can recognize this fault type by building an algorithm that finds the same address that stays in both transient and permanent fault queues. When an intermittent fault is found, it is listed in the permanent fault queue only and treated as a permanent fault.
**Algorithm 1** Collecting the error position from EDF to OS  1:**function**OS_MFM_collect_errors()  2:    **while** true **do**  3:        **if** EDF_Error_Found_Interrupt() **then**  4:           {Fault_type,addr}←EDF_get_fault_pos()  5:           **switch** Fault_Type **do**  6:               **case** TRANSIENT  7:                   transient_faulty_list.push(addr)  8:               **case** PERMANENT  9:                   permanent_faulty_list.push(addr)10:               **case** UNKNOWN11:                   unknown_faulty_list.push(addr)12:        **else**13:           sleep()14:**procedure**EDF_inform_error_to_OS_MFM()15:    **while** curr_cmd.cmd=READ **do**16:        **if** err_flag_triggered() **then**17:           addr←extract_err_pos(curr_cmd.address)18:           Send_faulty_pos(UNKNOWN,addr)19:        **else**20:           do_nothing()

#### 5.2.2. Error Diagnostic and Repair Procedure

After obtaining *unknown* errors, we can use the diagnostic and repair procedure, summarized in Algorithm 2. In this procedure, we first reread the faulty DRAM word and observe the corresponding BF. Then, based on the BF, we make the following actions.

- When the BF is 0×0000, we consider that the corresponding DRAM word has intermittent faults sent to the *permanent* fault queue. This is because, in the previous read, errors are reported from this DRAM word; however, no errors are found at this reading.
- When the BF is 0×00FF, it is clear that a multiple-bit error is detected and uncorrected, namely, DUE, by on-die ECCs. The DRAM word to cause the multiple-bit error is not categorized as *transient* or *permanent*. We only report the occurrence of multiple-bit errors to both the OS and the memory controller, handled by a rank-level ECC or other system-level techniques.
- When the BF has a single bit raised, regarded as *default*, or is 0×FFFF, we assume that the corresponding DRAM word has a single-bit fault in data or parity, respectively. Then, we call the function to handle single-bit faults; the corresponding algorithms are discussed as follows.

**Data Error Correction** We implement a function to repair faulty words stored in DRAM cells by using on-die ECCs, namely, *data error correction* (DEC), described in Algorithm 3. The repair can be obtained for the case of single-bit transient errors. When a faulty word with a single-bit transient error is read, on-die ECCs correct the error, and then one corresponding BF is enabled in our OBET architecture. Note that there are two more error cases in which one BF is raised: miscorrection due to multiple-bit errors and a single-bit intermittent or permanent error. For simplicity, the latter is referred to as a permanent single-bit error in this section since both intermittent and permanent errors are treated equally in this work. In conventional DDR5 DRAMs, on-die ECCs equally recognize the above three cases as single-bit errors of the read data and flip the bit regarded as erroneous. However, they do not fix the faulty DRAM cell that caused the error. Algorithm 3 clarifies which case occurs and repairs the faulty DRAM cell for the single-bit transient error case, where the following two sequences are required. First, we write back the read data that have already been fixed by on-die ECCs. Note that we do not update the corresponding parity bits at the writing-back operation. This can be obtained by exploiting BFs as the control signals, explained in Section 4.2.1. Finally, we read the written data again and checked the BF to ensure that no more errors occurred. Here, one of the following five cases potentially occurs.

- For the single-bit transient error, the read-out data are the same as the write-back data with no BF bits raised.
- For the single-bit permanent error, the read-out data are the same as the write-back data with one BF bit raised.
- For the miscorrection due to multiple-bit errors, the read-out data are different from the write-back data after the writing back, since a new error bit is created by on-die ECCs, which aim to correct wrong bit locations.
- For miscorrection due to multiple-bit errors, the read-out data are the same as the write-back data, while a notification of DUE is sent via BF.
- For miscorrection due to multiple-bit errors, the read-out data are the same as the write-back data with no BFs raised.

The faulty DRAM word to result in the single-bit transient error is repaired by the above sequences, and the address is pushed to the *transient* queue, while for the single-bit permanent error, the corresponding DRAM address is pushed to the *permanent* queue. For the third and fourth cases, notifications of multiple-bit errors are sent to both the OS and the memory controller. However, the last case is considered equal to the single-bit transient error, which cannot be fixed. We do not manage such a case since the corresponding probability is extremely low. Our OBET significantly alleviates the accumulation of single-bit errors, and hence, the probability is lowered further. Furthermore, this case can be significantly addressed with the support of a rank-level ECC. Even in the worst case, some system-level techniques, such as checksum, overcome this case.
**Algorithm 2** Diagnostic and repair of faults from the OS  1:**function**OS_MFM_diagnostic_fixing_errors()  2:    enable_request_for_diag_fix_err()  3:    **while** (addr←unknown_faulty_list.pop())≠null **do**  4:        Send_to_EDF(addr)  5:    disable_request_for_diag_fix_err()  6:**procedure**EDF_diagnostic_fixing_err()  7:    **while** OS_trigger_request_for_diag_repair() **do**  8:        addr←get_err_pos_from_OS()  9:        data←READ(addr)10:        type_flag←read_error_flag()11:        **switch** type_flag **do**12:           **case** 0×0000                  ▹ Intermittent Error13:               Send_faulty_pos(PERMANENT,addr)14:           **case** 0×00FF                    ▹≥2 bit errors15:               Alert_multiple_errors(addr)16:           **case** 0×FFFF               ▹ Parity has single error17:               Parity_err_correction(addr,data)       ▹ Algorithm 318:           **case** default            ▹ Original data have a single error19:               Data_err_correction(addr,data)        ▹ Algorithm 3

**Parity Error Correction** The function of parity error correction is similar to that of DEC. The only difference is that we need to allow the parity update when we write back the read data. The second read does not raise BF when the parity DRAM word has a single-bit transient fault, repaired by the write-back operation. However, when the BF of the second read still indicates that the parity bit has an erroneous bit, the corresponding DRAM word is sent to the *permanent* queue. Here, the miscorrection due to multiple-bit errors generates the illusion that a permanent faulty cell exists in the parity part, sent to the *permanent* queue. However, such a case has a very low probability, as mentioned above, handled by system-level techniques.
**Algorithm 3** Data/parity error correction  1:**procedure**Data_Err_Correction(addr,data)  2:    enable_error_flag()         ▹ Prevent Parity update  3:    WRITE(addr,data)      ▹ Corrected data from first READ  4:    disable_error_flag()         ▹ Allow Parity update  5:    diag_data←READ(addr)  6:    type_flag←read_error_flag()  7:    **if** diag_data≠data **then**  8:        Alert_multiple_errors(addr)  9:    **else**10:        **switch** type_flag **do**11:           **case** 0×FFFF‖0×00FF      ▹ DUE ≥ 2 bit errors12:               Alert_2_errors(addr)13:           **case** 0×000014:               Send_faulty_pos(TRANSIENT,addr)15:           **case** default16:               Send_faulty_pos(PERMANENT,addr)17:**procedure**Parity_Err_Correction(addr,data)18:    disable_error_flag()        ▹ Allow Parity update19:    WRITE(addr,data)20:    data←READ(addr)21:    type_flag←read_error_flag()22:    **if** type_flag=0×FFFF **then**     ▹ Permanent error23:        Send_faulty_pos(PERMANENT,addr)24:    **else**25:        Send_faulty_pos(TRANSIENT,addr)

#### 5.2.3. Remapping a Permanent Faulty Page

The on-die ECC only corrects the input data after they are read; it does not fix the actual hardware errors. Transient errors can be fixed by rewriting the correct data to the same location. Unlike transient errors, permanent errors cannot be fixed by rewriting, and they are troublesome during the running time of the system because they always cause bit errors and accumulate with other errors. This accumulation can easily crash the system. Hence, it is vital to eliminate permanent errors in the system. We can easily obtain the byte-error location in DRAM with the OBET and follow the error-correcting pointer (ECP) [38] to give the most efficient correcting method in DRAM. However, cache should be considered, and more hardware should be added to control the error-correcting pointer. To minimize such issues, we consider and follow the instruction of page retirement only [36,39] for a more flexible and straightforward technique that can be controlled in software/OS. The memory is mapped to the system as pages and frames. A memory management unit (MMU) manages such pages and frames. Typically, the MMU is initiated by the mapping data obtained from the hard drive when the system reboots. Hence, the MMU can reveal such mapping information (including faulty pages) during the rebooting time.

OBET can quickly adopt page retirement because permanent error locations have already been pointed out during the diagnostic procedure. In this technique, the corrected data on the page that has permanent error are remapped to a nonfaulty page, as shown in Figure 4c. This operation can be performed by the OS via the configuration of the MMU without affecting the multilevel caching system, as shown in Figure 4a.

## 6. Evaluation

This section presents our evaluation of OBET. We divide the evaluation into two main components. First, we measure the reliability enhancement provided by multiple OBET-based protection schemes when utilized with commercial protection schemes. For the second evaluation, we compare the performance and energy consumption of the OBET-based protection schemes with those of state-of-the-art approaches on the SPEC2006 benchmarks.

### 6.1. Multiple Types of OBET-Based Protection

To evaluate OBET when integrated with other protection schemes, we first review the commercial protection schemes that are widely used in current DRAM subsystems. We then discuss how to incorporate OBET with these commercial protection mechanisms in the DDR5 subsystem and evaluate these methods.

#### 6.1.1. SECDED

The simplest protection mechanism for a DRAM subsystem is SECDED. The proposed DDR5 architecture comprises 2 channels and 32-bit data for each channel. Because SECDED [40] requires 6 bits for parity checks, the JEDEC standard [18] requires 40 bits for each channel. When we apply SECDED(40,32), DDR5 enables a two-layered ECC. DDR5 with SECDED can correct one error in either the vertical or horizontal direction. If a double-bit error occurs in one direction (either vertical or horizontal), there is a high chance that the ECC of the other direction can correct both errors if they are located in two different bytes. However, SECDED corrects only a single-bit error and cannot correct multiple errors, while a group of errors is still in consideration in modern DRAM main memory [27].

#### 6.1.2. Chipkill-Correct

**Figure 5:**
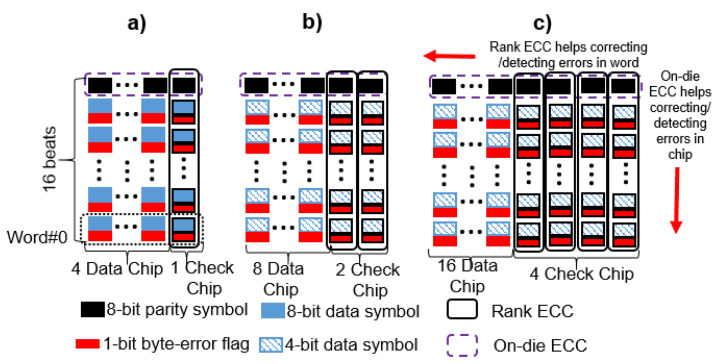
A visualization of all OBET-based techniques for the (**a**) SECDED, (**b**) Chipkill-SSC, and (**c**) Chipkill-SSCDSD schemes.

In the current DRAM architecture, both ×8 and ×4 configurations are widely used; ×8 configurations are preferred for power efficiency, and ×4 configurations are preferred to achieve higher reliability. To correct multiple errors in a device, AMD Chipkill architecture [41] provides two levels of Chipkill: (1) single symbol correction-double symbol detection (Chipkill-SSCDSD) and (2) single symbol correction (Chipkill-SSC). For Chipkill-SSCDSD, AMD requires 36 × 4 DRAM chips (32 DRAM chips for data symbols and 4 chips for check symbols) to protect the 64-bit data channel. To realize Chipkill-SSCDSD on DDR5 with a 32-bit data channel, assuming all bit locations of more than 32 bits become dummies, we need 16 data symbols and 4 check symbols. For the single symbol correction, Chipkill-SSC employs the Reed Solomon code on a 4-bit symbol. This requires 8 data symbols and 2 check symbols, which completely fits into the current ×4 DRAM configuration of DDR5. Note that the Chipkill-SSC and Chipkill-SSCDSD considered in our paper are already combined with on-die ECC. All comparisons with SECDED, Chipkill-SSC, and Chipkill-SSCDSD are shown in Figure 5.

#### 6.1.3. Merging the Alert_n Pin in a DIMM

With the support of OBET, which can expose on-die ECC errors to the system level, OBET, OBET-SSC, and OBET-SSCDSD correspond to the use of the SECDED, Chipkill-SSC, and Chipkill-SSCDSD protection schemes, respectively. To support a large number of data symbols and check symbols, our OBET-supported schemes require fine-grained and coarse-grained architecture. For the fine-grained architecture, the additional pins for Alert_n can be obtained by extending DIMM pins. Thus, this fine-grained architecture can provide byte-level error diagnostics. On the other hand, the DIMM module is not changed for the coarse-grained architecture because merging all Alert_n signals to one signal with a simple OR-gating is possible. However, coarse-grained architecture can perform diagnostics only for each burst in data transfers. Hence, there is a trade-off between choosing the diagnostic level (i.e., byte or word-level error) and extending the DIMM module.

### 6.2. Methodology

Based on the above discussion, we summarize the all different schemes in Table 1. These schemes are evaluated in terms of their reliability, performance, and energy consumption. To conduct a comparison between different OBET-based schemes, we perform a Monte Carlo simulation with pattern errors. A Monte Carlo simulation requires repeated random sampling to obtain the distribution of an unknown probabilistic entity [42]. By default, we use the pattern error models defined according to the failure-in-time (FIT) rate from Table 2, which present failures per billion hours for all elements in DRAM devices. We build simulations based on some tools [43] and then implement these simulations by injecting a failure at a certain point in time based on the FIT rate for 7 years. Error correction is invoked periodically in each scheme by reads or at the scrubbing interval to determine the detection, correction, and uncorrectable rates of these schemes. While running 100 million experiments, we count the number of experiments that stop working due to uncorrectable errors [19] to enable a comparison with other works. In our simulations, we perform 2 types of error elimination: (1) eliminating transient errors and (2) eliminating transient or permanent errors every 24 h. Apart from OBET-based corrections, we also evaluate and compare OBET with XED [19], DUO [20], and PAIR [35], which were introduced to enhance the reliability of DRAM. We perform the simulations and show the reliability comparison between OBET and state-of-the-art works such as XED [19], DUO [20], and PAIR [35] with commercial DRAM protection schemes such as SECDED, Chipkill-SSCDSD, Chipkill-SSC, and OBET-based protection schemes. To facilitate comparisons with the state-of-the-art works, we consider the ×4 DDR5 configuration and evaluate all correction schemes, as shown in Table 1.

To conduct performance and energy consumption comparisons with the state-of-the-art works, we simulate the performance and energy consumption of OBET-based correction schemes relative to those of the above state-of-the-art works. We run the SPEC2006 [44] benchmarks on GEM5 [45] with an Intel D-1649N x8 CPU [46] and DDR5 DRAM. For DDR5, we use 2 channels of DDR5 DIMMs implemented with 10 chips and 4-bit chips with a data rate equal to 3200 MHz. The DDR5 timing model is taken from JEDEC79-5 [18], and the power model is taken from DDR4 [47]. For SPEC2006, we form 3 groups based on memory usage: low, medium, and high memory usage. We perform 7 simulations, involving (1) normal DRAM, (2) SECDED, (3) XED, (4) DUO, (5) PAIR, (6) Chipkill-SSC, (7) Chipkill-SSCDSD, and normal DRAM, which is considered the baseline in this simulation. For XED, we estimate the delay of the XOR operation by using a low-power 65-nm CMOS. The time cost is 0.57 ns. For DUO, we modify GEM5, which can support a burst length of 17, and evaluate the latency of Reed-Solomon(76,64) based on [48,49]. We extract the encoding/decoding latency depending on the data rate of the DDR DIMM. The encoding/decoding latency is 0.94/67.8 ns at 3200 MHz. Similar to the DUO case, we evaluate the latency of Reed-Solomon(40,32) for PAIR. The encoding/decoding latency is 0.94/48.4 ns at 3200 MHz. For SECDED, we use SECDED for rank-level ECCs with an estimated latency equal to 3.04 ns. Furthermore, we modify GEM5 to support reading, modification, and writing with a writing latency that is four times greater than the reading latency yielded by normal DRAM, XED, SECDED, Chipkill-SSC, and Chipkill-SSCDSD. All configurations are summarized in Table 3.

### 6.3. Evaluation Results

#### 6.3.1. Enhanced Error Detection

**Figure 6:**
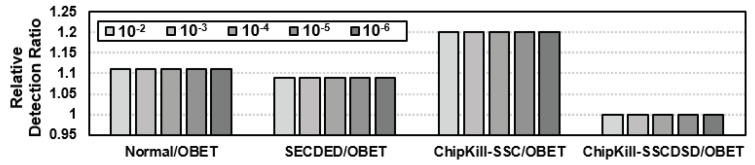
Error detection rates are significantly increased with the support of OBET on various bit-error rates (BERs).

Regarding their error correction capacities, OBET-based correction schemes are similar to non-OBET-based schemes. However, the important contribution of OBET is to expose the internal errors to rank-ECC. This can significantly increase the number of error detections at rank-ECC. When single errors occur, OBET can expose these errors and help the system eliminate such errors by scrubbing or replacing them. When double errors occur in the same codeword in DRAM, they can be easily fixed. However, correcting double errors remains an open problem without OBET. By injecting uniform random errors and considering the error accumulation to the ×4 DDR5 configuration, we achieve 11%, 9%, and 20% errors on normal, SECDED, and Chipkill-SSC, respectively. The detection is not improved as much for Chipkill-SSCDSD because it already uses a very efficient detection technique. The results are shown in Figure 6.

#### 6.3.2. Reliability Comparison with State-of-the-Art Works

**Figure 7:**
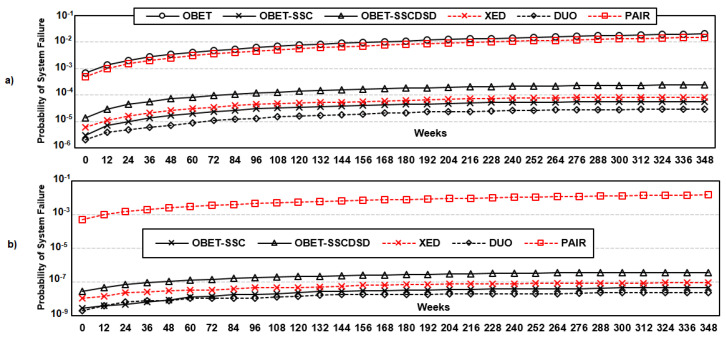
The probability of system failure for state-of-the-art works and OBET-based techniques for a period of 7 years based on Monte Carlo simulations with pattern error [27]: (**a**) removal of transition errors only and (**b**) removal of both transient and permanent errors.

The results obtained with DDR5 are shown in Figure 7. DUO shows the highest reliability compared to the other protection schemes because it can correct up to six symbol errors or one device and one symbol error, but the drawback is a large overhead for decoding Reed-Solomon(76,64). SECDED can correct only single-bit errors, while multiple-bit errors also occur, as reported in [27]. PAIR is a restructured DRAM architecture to reduce the overhead of ‘read-modify-write’ on write operation; it corrects up to 4 symbol errors due to Reed-Solomon(40,32), and tolerant multiple-bit errors occur while the approach is still vulnerable to device-level errors. For the device-level errors, both SECDED and PAIR show the lowest reliability in DDR5. OBET-based protection schemes are more reliable than XED and less than 1.5∼2 times as reliable as DUO. However, OBET-SSC and OBET-SSCDSD can guarantee every 32-bit burst data instead of the whole block of data (i.e., 512 bits) as with DUO and PAIR, while the overheads are considerable.

#### 6.3.3. Performance and Energy Consumption Comparison

**Figure 8:**
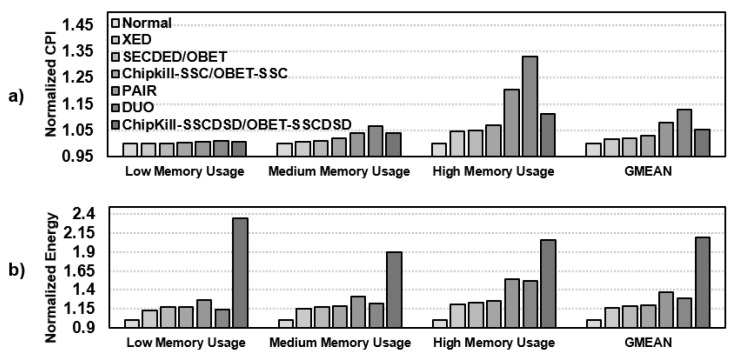
Simulation results for OBET-based techniques and state-of-the-art works on various applications: (**a**) normalized cycles per instruction (CPI) and (**b**) normalized energy consumption.

The performance results are summarized in Figure 8a. Without any error, SECDED, Chipkill-SSC, and Chipkill-SSCDSD achieve the same performance as the OBET-based protection schemes. XED, SECDED, Chipkill-SSC, and Chipkill-SSCDSD induce only minor 0.2∼3% performance degradation compared to normal DRAM. However, DUO and PAIR have a significant performance degradation due to the large overhead of RS(76,64) and RS(40,32), at approximately 8% and 12.8% degradation, respectively.

By utilizing double the number of DRAM chips, Chipkill-SSCDSD increases energy consumption by more than 2.1× compared to that of normal DRAM. XED, SECDED, and Chipkill-SSC energy consumption are mostly similar and increase the energy consumption by more than 16∼20% compared to normal DRAM. PAIR and DUO show increases of 36.8% and 28.4% compared to normal DRAM, respectively, due to the large overhead of Reed Solomon code on long codewords. All the experimental results are shown in Figure 8b.

## 7. Efficient OBET-Based Scrubbing

Scrubbing is a popular technique that eliminates transient errors by writing back the correct data to the erroneous location. However, the system manager must perform the scrubbing process for every given scrubbing interval (i.e., 24 h). Unlike the ECS mode in DDR5, which requires all code words in DRAM to be written back, the system manager can select the error codeword for scrubbing based on byte-level error information. As a result, OBET can replace the conventional scrubbing technique. With the support of OBET, we can eliminate both permanent and transient errors with fewer cycles during scrubbing. Furthermore, OBET scrubbing checks only whether the error occurred inside the DRAM chip. Hence, OBET scrubbing can easily adapt to coarse-grained architecture without modifying the DIMM module. Although the fine-grained architecture requires DIMM modification, it is very helpful in industry for debugging purposes.

### 7.1. OBET Scrubbing

With technology scaling, the error rate tends to increase. Thus, the JEDEC standard [18] requires that the scrubbing be operated at least once every 24 h. The large number of scrubbing codewords ($2^{26}$∼$2^{29}$ codewords) internally follow the sequence ACT, RD, WR, and PRE. This ECS mode is inefficient because it is performed on the whole DRAM device every 24 h. Furthermore, server downtime due to scrubbing is a considerable problem. However, we need to scrub only the unaccessed DRAM space of the OS-allocated space. Hence, some DRAM spaces do not need scrubbing, such as (1) unused DRAM spaces and (2) the DRAM spaces already accessed within 24 h. To solve such issues, our OBET scrubbing can operate efficiently with a lower number of codewords. In summary, OBET scrubbing targets (1) fix the unexposed error for each accessing request to the accessing space and (2) selectively perform scrubbing on unaccessed spaces of allocated DRAM spaces every 24 h. The algorithm of OBET scrubbing is shown in Algorithm 4.
**Algorithm 4** OBET-scrubbing  1:**function**OS_OBET_scrubbing()  2:    **while** true **do**  3:        **if** scrubbing_counter≤0 **then**  4:           **for each** addr∈OS **do**  5:               **if** $\left(addr.is\_not_{\_}accessed\_within\_24hrs\right)$ **then**  6:                   READ(addr)       ▹ Read only and collect errors  7:           enable_request_for_diag_fix_err()  8:           **while** (unknown_faulty_list.is_not_empty()) **do**  9:               addr←unknown_faulty_list.pop()10:               Send_to_EDF(addr)         ▹ Algorithm 211:           disable_request_for_diag_fix_err()12:           scrubbing_counter←2413:        count_down_hours(scrubbing_counter)

**Figure 9:**
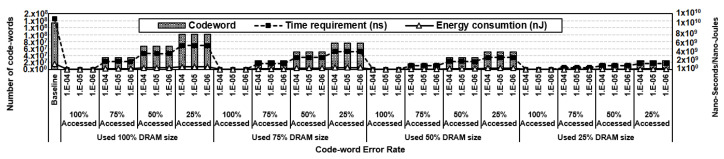
Scrubbing codewords, scrubbing time, and scrubbing energy evaluation results of OBET scrubbing on 16 GB DDR5.

In OBET scrubbing, single-bit errors can be easily tracked at the byte level. Based on the type of error, each of them can be processed by different strategies: (1) most of the time, when no errors occurred, we only read the data and check the Alert_n pin; (2) for transient errors, we simply write back the read data for correction; and (3) for permanent errors, we map the read data to another nonfaulty location with an addition write command to a new location. The sequences of OBET scrubbing are described in Table 4. The main difference between OBET scrubbing and DDR5’s ECS is that instead of performing the read and write back for all codewords in ECS mode, we selectively perform a scrubbing procedure customized to the type of error. To compare the efficiency of OBET scrubbing to that of DDR5’s ECS mode, we estimate the OBET scrubbing overhead on 16 GB DDR5 based on [50] with the timing and current parameters shown in Table 3. Compared to ECS, our estimation shows that OBET scrubbing can significantly reduce the number of codewords because of the small codeword error rates. Similar to the ECS mode, OBET scrubbing incurs high overhead only when the codeword contains an error. Otherwise, more than 99% of the time, the overhead is simply a read operation compared to both read and write-back in ECS. Further scrubbing time and scrubbing energy are significantly reduced in OBET scrubbing, with the details shown in Figure 9.

### 7.2. Eliminating Errors with OBET Scrubbing

**Figure 10:**
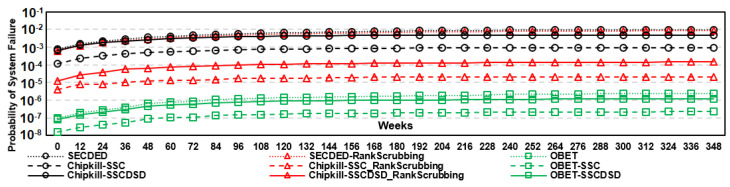
The probability of system failure over a period of 7 years based on uniform random errors: a comparison between commercialized cases and OBET scrubbing.

To show the impact of OBET scrubbing that enhances the reliability of DDR5-based correction schemes, we inject uniform random errors with the single bit failure rate, as shown in Table 2, and increase the error factor to 100 times to demonstrate the predicted $10^{-6}$∼$10^{-4}$ bit-error rate [21]. In this simulation, we consider only that the error is a random flipping of the data stored in the DRAM cell in terms of transient and permanent errors. We consider three cases: (1) ECS mode of DDR5, in which only the transient errors can be eliminated every 24 h, (2) both transient and permanent errors caught by the rank-ECC can be eliminated, as supported in Mcelog [36,39], and (3) OBET scrubbing can eliminate any single-bit errors that are caught from OBET and rank-ECC. Case-1 and case-2 are applied for normal error protection schemes, and case-3 is our OBET-based scheme, where unexposed errors can be tracked on the fly. As shown in Figure 10, the system reliability can improve 5000∼7000× with OBET scrubbing compared to the ECS of DDR5. We note that without exposing the error inside DRAM cells, the reliability of systems is significantly degraded.

### 7.3. On-the-Fly Error Correction with OBET

**Figure 11:**
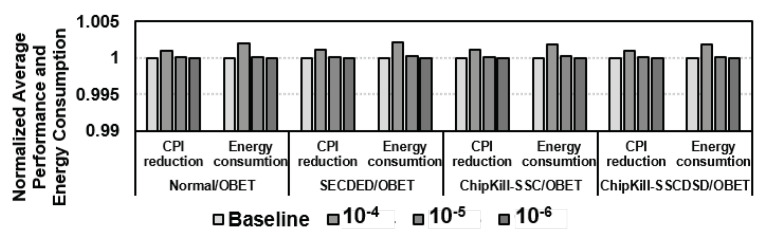
Comparison of performance and energy consumption with different codeword error rates.

To achieve more efficient scrubbing time and energy, we can exploit on-the-fly error tracking to correct the error codewords. We directly correct the unexposed errors during run-time instead of waiting for some interval, as discussed earlier. We modify GEM5 to support OBET that can catch the errors during run-time, and we assume that all the errors will become permanent errors that require replacing a page (typically 4 kB) by 66 READ and 65 WRITE, as shown in Table 4. We perform the simulation with different codeword error rates by taking the average performance and energy impact on SPEC2006. As shown in Figure 11, the overhead of on-the-fly error correction is negligible compared to the related works. Here, we consider normal DRAM, SECDED, Chipkill-SSC, and Chipkill-SSCDSD without error for comparison.

## 8. Conclusions

We have presented OBET, a DRAM architecture that can track byte-level or half-byte-level errors with minimal overhead. The key premise of OBET is exposing the error information from the on-die ECC to the OS for error correction and detection purposes. After classifying the errors into temporary or permanent errors, we apply scrubbing and remapping of target faulty pages. By doing so, our evaluation shows that OBET-based schemes can achieve near-level reliability of state-of-the-art solutions while negligible performance and energy consumption. As byte-error information is available to the OS level, we expect OBET to enable smart and more efficient software-based error correction solutions in the near future, especially when technology scaling makes bit errors more severe.

## Figures and Tables

**Table 1:** DDR5 correction schemes per channel.

DRAM Conf.	DDR5 Correction Schemes	Num. of Data/Parity Chip	Check Code	Latency Cycles (ns@3200 MHz)	OBET- Scrubbing
X8	SECDED	4/1	SEC+SECDED	1.5 ns	
	OBET	4/1	SEC+SECDED	1.5 ns	X
	XED [19]	4/1	XOR	0.5 ns	
X4	SECDED	8/2	SEC+SECDED	1.5 ns	
	OBET	8/2	SEC+SECDED	1.5 ns	X
	XED [19]	8/1	XOR	0.5 ns	
	DUO [20]	8/1	RS(76,64,8)	217(67.81 ns)	
	PAIR [35]	8/2	RS(40,32,8)	123(38.43 ns)	
	Chipkill-SSC	8/2	RS(10,8,4)	35(10.94 ns)	
	OBET-SSC	8/2	RS(10,8,4)	35(10.94 ns)	X
	Chipkill-SSCDSD	16/4	RS(20,16,8)	61(19.06 ns)	
	OBET-SSCDSD	16/4	RS(20,16,8)	61(19.06 ns)	X

**Table 2:** Failure rate of each elements in DRAM devices per billion hours (FIT) [27].

Failure Mode	Failure Rate (FIT)	
	Transient	Permanent
Single-bit	14.2	18.6
Single-word	1.4	0.3
Single-column	1.4	5.6
Single-row	0.2	8.2
Single-bank	0.8	10.0
Multiple-bank	0.3	1.4
Multiple-rank	0.9	2.8

**Table 3:** Evaluation configuration of OBET-based schemes and the state-of-the-art works.

CPU	Intel D-1649N x8-3 Ghz
System configuration	System bus: 1 GHz
	L1-I/L1-D: 32 kB|L2: 256 kB|L3: 12 MB
	64-byte cache line—2/8/16-ways
DRAM DDR5	16 Gb DDR5_DIMM_×4
	Clock/data rate: 1600 MHz/3200 Mbps/pin
	2 channels/ FRFCFS scheduler
	Timing models: JEDEC79-5 [18]
	Energy models: GEM5
ECC latency(ns)	Table 1
Benchmark	**Low**: perlbench, hmmer, libquant,
	namd, omnetpp, tonto, povray, sjeng
	**Medium**: gamess, gromac, sphinx3,
	wrf, astar, zuesmp, calculix, leslie3d
	**High**: gcc, bzip2, bwaves, milc,
	gobmk, lbm, mcf

**Table 4:** Scrubbing sequence comparison between conventional ECS mode and OBET scrubbing.

Scrubbing Mode	Sequence	Note
DDR5 ECS mode	ACT→RD→WR→PRE	All codewords
OBET scrubbing	ACT→RD→PRE	No error codewords
	ACT→RD→WR→RD→PRE	Transient errors only
	ACT→RD→WR→RD→64(RD) →ACT→64(WR)→PRE→PRE	Permanent errors only
